# Unpacking the pathways using the stress and coping theory: How social support reduces substance abuse through depression

**DOI:** 10.1097/MD.0000000000050725

**Published:** 2026-09-18

**Authors:** Kelemu Zelalem Berhanu, Kefie Manaye Mengistie

**Affiliations:** a University of Johannesburg, Johannesburg, South Africa; b Department of Psychology, Debre Markos University, Debre Markos, Ethiopia.

**Keywords:** depression, higher education, social support, substance abuse

## Abstract

Substance abuse among university students is a significant public health concern that is typically linked to underlying mental health issues such as depression, stress, and a lack of social support networks. Thus, the study’s goal was to investigate the link between social support, depression, and substance abuse. A cross-sectional design was used. Students were given 3 measures to complete for the current study: the Multidimensional Scale of Perceived Social Support, the Beck Depression Inventory, and the Alcohol, Smoking, and Substance Involvement Screening Test. The study included 257 regular undergraduate students from Addis Ababa Institute of Technology. As a result, the multivariate test of significance demonstrates that parents’ marital status, monthly income, and the presence or absence of intimate friends all have a significant influence on student substance abuse and depression. The Pearson correlation demonstrated a substantial positive relationship between depression and substance abuse; however, higher levels of social support are associated with a decreased likelihood of substance abuse and depression among students. Finally, depression has a mediating role in the relationship between social support and substance abuse. These findings suggest that investing in supportive relationships and communities might be an effective way to improve mental health while simultaneously lowering risky behavior like substance abuse. Furthermore, the researchers proposed that the institution establish its own substance abuse prevention and treatment center, available to psychologists, therapists, and other health practitioners.

## 1. Introduction

University student substance abuse is a growing public health issue that is frequently associated with underlying mental problems including depression and a lack of social support systems. Depression is one of the main causes of sickness in young adults, and the hazards to one’s health are greatly increased when it coexists with substance abuse.^[1]^ Lazarus and Folkman^[2]^ stress and coping theory provide a useful framework for comprehending the psychological processes that underlie the relationship between social supports, substance abuse and depression.

Social support is a vital coping mechanism in this situation, mitigating the negative impacts of depression and lowering the risk of psychological discomfort. After controlling for alcohol consumption, depression was substantially linked to both moderate and perceived low social support.^[3]^ People drinking and depression can be significantly impacted by peer groups. People who start drinking too much alcohol at a young age and later join a deviant social group may be especially vulnerable to depression in the future. By associating with deviant friends, depressed mood also predicted harmful drinking.^[4]^ An elevated likelihood of incident mental issues like depression is independently linked to substance addiction distress and friendship dissatisfaction. According to an integrative perspective, social support can indirectly lower the likelihood of substance abuse by lowering psychological distress and depression. Social support acted as a buffering variable in one study, although psychological distress was found to modulate the association between substance use and maltreatment during childhood. These results highlight the significance of establishing robust support networks in educational settings in order to stop the negative feedback loop between depression and substance abuse.^[5]^

According to existing studies, adolescents who receive less parental support are more likely to use drugs and exhibit negative feelings.^[6]^ Research on students has found that parental support protects against a variety of outcomes, including alcohol and other drug use, depression, and anxiety.^[7]^ However, it has been demonstrated that while strong social support is a protective factor against substance use, poor parental support is related with higher levels of substance use.^[8]^ Despite substantial proof of social support’s protective function in mental and physical health; significant gaps exist in our comprehension of the routes and mediation mechanisms that underpin these associations in certain vulnerable populations. Social support is unquestionably important for both mental and physical wellbeing. Social support has been shown to have a direct (main impact) association with mental health, including psychological depression and substance abuse.^[9–13]^

The present study fills numerous major research gaps in the existing literature. First, while stress and coping theory^[2]^ is commonly utilized in Western educational settings, there is very little information on how these variables work in Ethiopian higher education institutions, where cultural, economic, and institutional contexts can significantly alter the relationships between depression, social support, and substance abuse. Furthermore, the particular routes by which various sorts of social support, such as emotional, instrumental, or peer-based, buffer depression and, as a result, prevent substance use remain unexplored in this context. Another gap lies in determining whether depression is an entire or partial mediator in the relationship between social support and substance abuse, as well as how socioeconomic status affects students’ level of depression and substance abuse. Likewise, Ethiopian universities face unique stressors such as workload, ethnic conflict, monetary constraints, and inadequate resources for mental wellness, but little is known about how support systems (family, peers) interact with formal university services to reduce substance abuse and depression. Finally, there is a scarcity of evidence-based therapies specific to Ethiopian higher educations that employ social support to address depression-related substance abuse. This study’s investigations into these gaps add to both theoretical and practical understandings of depression, social support, and substance abuse in a non-Western academic setting. Filling these gaps might assist Ethiopian universities in developing stronger support systems that prevent substance misuse by addressing psychological issues in a more committed, culturally compatible fashion. Therefore, the study’s goal is to investigate the function of depression in mediating the association between social support and substance abuse at Addis Ababa University in Ethiopia. To achieve this goal, researchers posed the following study questions:

1. Are socio-demographic factors having significant effect on the combined dependent variables (depression and substance abuse)?
2. What is the correlation between social support, depression, and substance abuse?
3. What is mediating role of depression in associations between social support and substance abuse?

## 2. Review literature

### 2.1. Social support and depression

Social support is an important aspect in preventing depression among students. Students with low-quality social support are a 6-fold increase in the likelihood of depressive symptoms as compared to those with adequate assistance. Perceived social support, which comprises emotional, informational, and instrumental aid, serves to mitigate the adverse effects of academic and social challenges by increasing students’ psychological resilience.^[14]^

The complex idea of social support is assessed in a variety of contexts, including friends, relatives, and another person of significance. It is believed to protect mental health both directly through the benefits of social contacts and indirectly by functioning as a buffer against stressful events.^[15]^ According to Bourne et al,^[16]^ a lack of social support might contribute to emotional tiredness. In addition to minimizing the outcomes of stressful events, social and emotional support may enhance coping with diseases and risk factors such as drinking and smoking.^[17]^

The quality of social relationship has a greater impact on the degree of depression.^[3,18–20]^ As a result, adolescents with supportive, close, and stable connections and friendships report fewer interpersonal conflicts and lower levels of depression.^[19]^ According to Sung-Min and Byoung-Jin,^[20]^ friendship is vital for maturing and improving your social and interpersonal abilities and capacities. Other findings from studies suggested a moderate and negative significant relationship between social support and depression in teenagers.^[21,22]^

In Ethiopia, social support plays a vital role in protecting young people – especially university students – from depression. When students feel supported by their family, friends, or peers, they tend to cope better with the pressures of university life. A study at Dilla University, Ethiopia, for example, found that students who felt they had little or no support were much more likely to experience moderate to severe depression.^[23]^ Another study at Debre Markos University, Ethiopia showed that emotional support was just as important as financial stability in helping students manage stress and avoid depressive symptoms.^[24]^ In Ethiopian culture, strong familial and social relationships are usually an expression of warmth and courage. However, for many students, particularly those studying away from home, strengthening social support within universities might be an effective method to help students feel less depressed and more resilient toward emotions.

### 2.2. Social support and substance use

Another important factor influencing substance use habits in higher education is social support. Family and peer interactions that are supportive are protective in nature and frequently prevent the adoption of unhealthy coping strategies, such as substance abuse.^[14]^ According to Ünver and Gül^[25]^ study of Turkish health profession students, substance usage and propensity for substance misuse rose as perceived social support declined. Environmental factors include social settings and community attitudes, peer influence, and a lack of other options for enjoyment and diversion increase the likelihood of substance addiction, according to Kebede et al.^[26]^ Tobacco, alcohol, and drug use are all predicted by peer context, according to a wealth of data.^[27–31]^ Additionally, there is a strong positive correlation between teenage substance addiction and social support. Teenagers choose their classmates according to social group norms, and over time, the peer group may be conditioned to conform. Adolescents may, for instance, associate with their substance-using classmates because they perceive substance use as the standard and may change their conduct to fit this social context.^[32]^

Adolescent substance usage is positively correlated with peer popularity perception. Compared to relationships between drug use and other peer-connectedness factors, this one is stronger and more persistent, highlighting the need for these constructs to be operationalized precisely and explicitly^[27]^. In settings where peer-connecting social outlets (such as clubs, sports teams, and friendship groups) also offer easy access to drugs, a positive link might be anticipated.^[27,28]^ The relationship between adolescent substance use and peer connection is likely to be overestimated if peer substance usage is not taken into consideration.

### 2.3. Depression and substance abuse

There is a well-established, reciprocal association between substance addiction and depression. Cannabis and tobacco use were substantially linked to depressed symptoms among Canadian postsecondary students, suggesting a propensity to self-medicate psychological suffering.^[33]^ This connection was further supported in varied student populations by a study conducted among Nigerian medical students, which discovered a substantial correlation between alcohol and psychoactive substance usage and depressed symptoms.^[34]^

Substance misuse can cause new mental health diseases or worsen preexisting ones, and it is strongly associated with mental health illnesses. Among the effects on mental health are: Increased risk of depression and anxiety: Abuse of substances can cause or exacerbate depressive and anxious symptoms, frequently resulting in a vicious cycle of substance use and mental health problems.^[35,36]^ The definition of depression includes feelings of gloom, rage, melancholy, or unhappiness. When someone’s depressive symptoms substantially disrupt their daily activities, they are said to have clinical depression.^[7,37]^ Stressors include parental substance abuse, parental separation or divorce, a family member’s sadness, or feelings of inadequacy can also trigger it.^[7]^ Melancholy brought on by these circumstances has been cited by several youths as a driving force behind their decision to begin drug usage. This type of “self-medication” is commonly used by teenagers who suffer from depression but may not be considered clinically depressed.^[38]^

Depression and drug use problems often co-occur in adolescents, and studies have shown a link between these 2 outcomes.^[39]^ There is evidence to show that teens who are depressed may be more susceptible to develop a drug use issue early in life. According to Joel research, mental health conditions at baseline presented substantial potential risks for the development of alcohol, nicotine, and illicit substance dependence and abuse over the follow-up period.^[40]^ Particular mood and anxiety disorders, behavioral disorders, history of substance use, and other ailments were found to have significant and enduring associations^[21,41]^

### 2.4. Theoretical framework

The stress and coping theory, which was put out by Lazarus and Folkman in 1984, has important and complex ramifications for comprehending students’ well-being in higher education environments. According to Lazarus and Folkman,^[2]^ the stress and coping theory has supported studies on the wellbeing of students in higher education. Lazarus and Folkman^[2]^ stress and coping theory has important and complex ramifications for comprehending how well students are doing in higher education environments. According to this Theory, depression is not only a response to outside forces like demands on one’s time at university or changes in one’s life; rather, it is greatly influenced by one is appreciated and supported by others-whether from family, peers, or institutional sources. Nevertheless, students who encounter loneliness or lack assistance may view life and academic obstacles as too much to cope with, which can result in signs of depression.^[42,43]^

**Figure 1.**
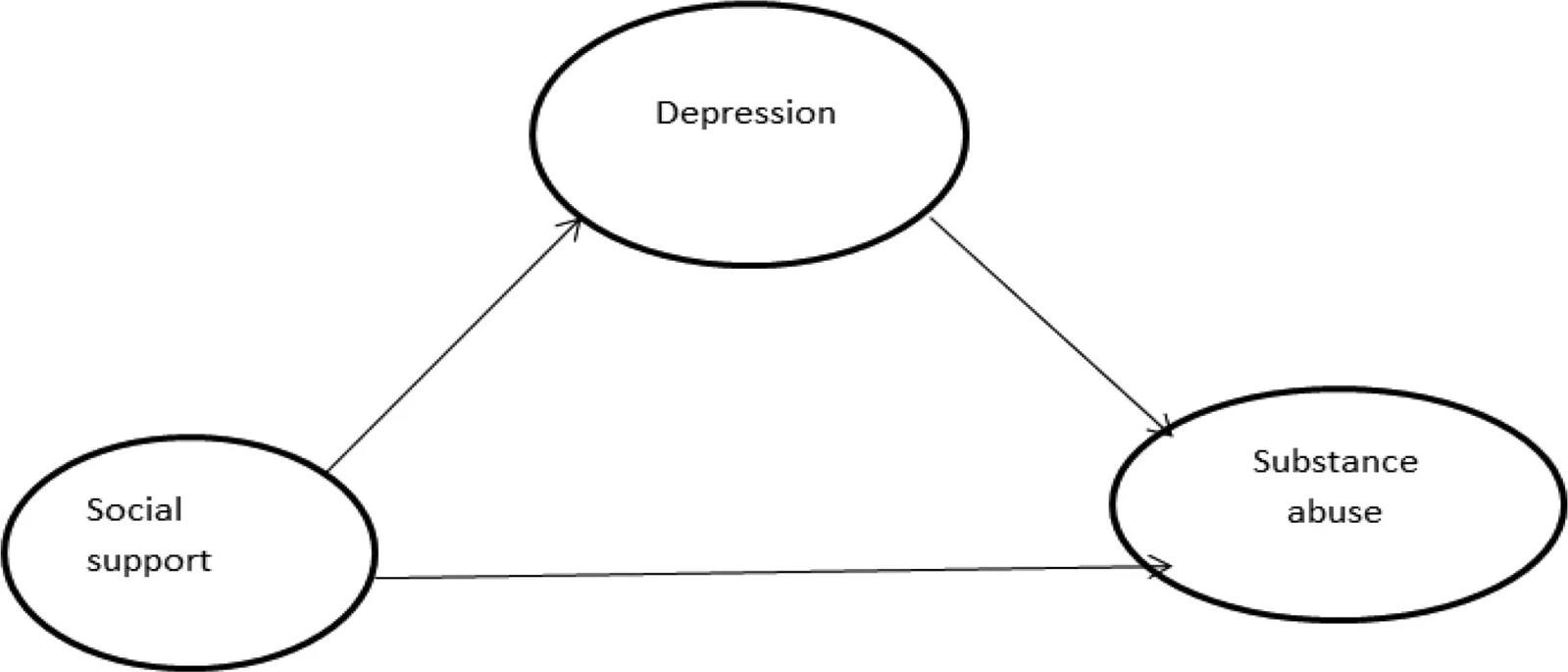
Conceptual framework of the study. This framework illustrates the hypothesized relationships between the key variables investigated. It outlines the interaction between independent variables (e.g., social support), mediating factor (depression), and the dependent variable(s) (e.g., substance abuse). The arrows indicate the direction of expected influence based on theoretical foundations and prior research.

Adapting stress and coping theory to learners in educational institutions offers a helpful structure for examining the way that social support influences hazardous habits like substance abuse as mediated by psychological issues like depression. According to the Theory, adolescents may see personal or academic challenges as overwhelming when they don’t feel supported, which might result in elevated depression levels. Adolescents may be more likely to take substances as an inappropriate method of coping depressed mood. The relationship between substance abuse and low perceived social support is made clearer by this approach, which includes student depression as a mediator ingredient. Enhancing social support, whether via family ties, peer interactions, or the support of institutions, may assist lessen depression and, in turn, indirectly lower the likelihood of using substances. Ultimately, the theory provides a thorough and nuanced approach to enhancing the psychological well-being of higher education students, emphasizing the importance of emotional support networks and constructive coping mechanisms in fostering resilience and averting negative behaviors in educational settings. Based on Lazarus and Folkman^[2]^ stress and coping theory, the conceptual framework depicted in Figure 1 shows students’ psychological wellness is protected by perceived social support, and its influence on substance abuse is mediated by depression.

## 3. Methodology

### 3.1. Research design

The present study employed the correlational study approach to build a general framework that integrates plenty of variables under various circumstances. It is essential to note that associations between parameters may not always indicate causality.^[44]^

### 3.2. Population, sample, and sampling techniques

**Figure 2.**
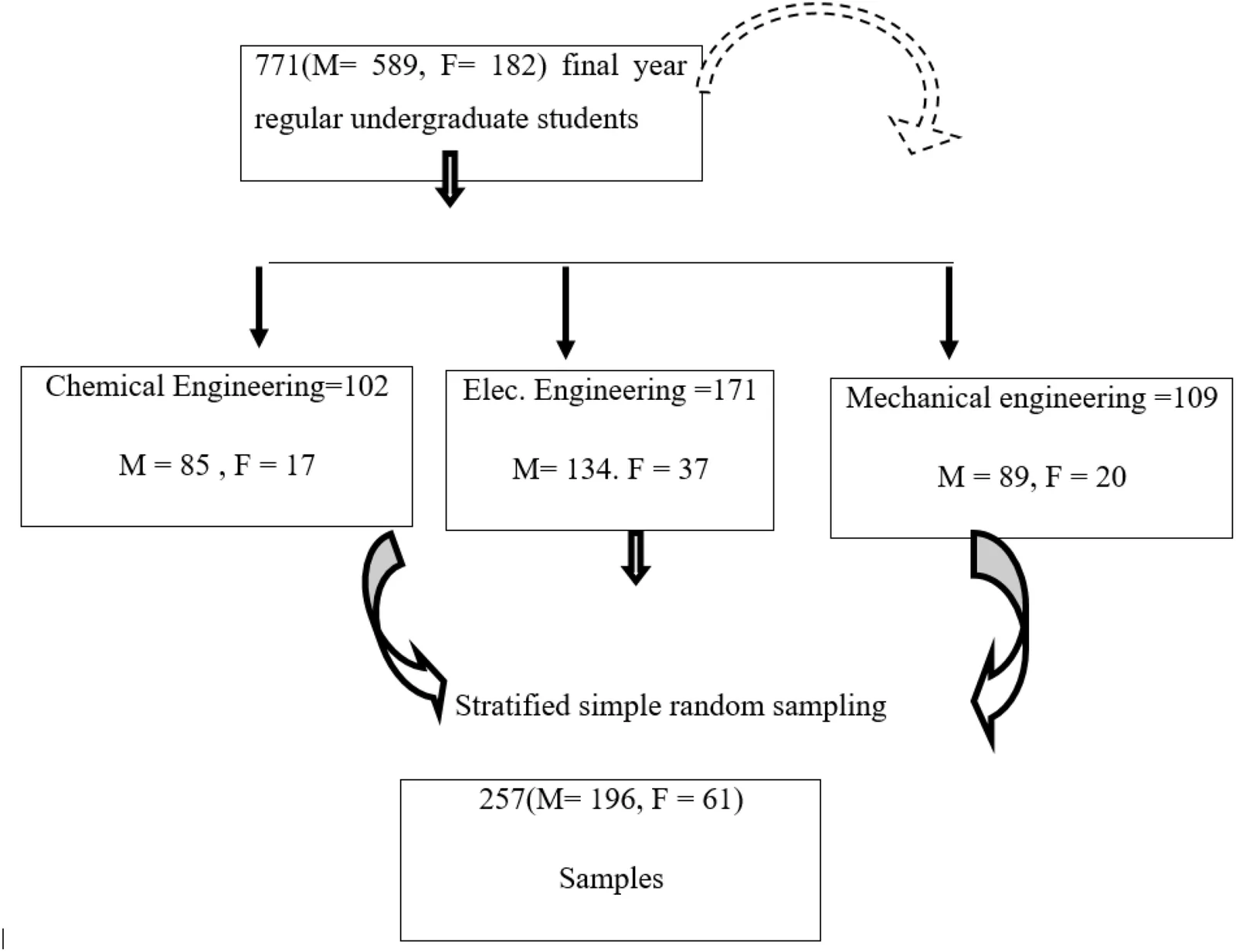
The flow diagram showing how final-year undergraduate engineering students were chosen for the research. Seven hundred seventy-one students (M = 589, F = 182) from 3 departments were initially taken into consideration: Mechanical Engineering (n = 109; M = 89, F = 20), Electrical Engineering (n = 171; M = 134, F = 37), and Chemical Engineering (n = 102; M = 85, F = 17). There were 257 students in the final sample (M = 196, F = 61). The dashed arrow denotes a backup route taken into consideration during recruitment, while solid arrows show the flow of participants from the entire pool through each school. M stands for male, F for female, and n for the total number of participants.

The study had been carried out at Addis Ababa University, specifically its Institute of Technology. The study site was selected purposefully because of the close nature of the institution to the researchers’ home and working place. The target population was Institute of Technology, which has a total of 6758 regular undergraduate students divided into 5 batches. Undergraduate students participated; consequently, summer and distance students at Addis Ababa Institute of Technology were not all available at the same time as the data collection. From this target the population, 771 final-year undergraduate students from 4 engineering schools were chosen as study population. Among 4 schools, 3 of the schools were randomly selected and stratified. The study population included 182 female students (23%), and 589 male students (77%). Then, using Krejcie and Morgan^[45]^ calculation with 95% confidence level and 5% marginal error, 257 students were picked. The sample size of 257 was statistically sufficient for the intentional inferential analyses, as it surpasses generally suggested edges for distinguishing medium effect sizes (*f*^2^ ≈ 0.15) with a predictable statistical power of 0.80 at α = .05, in terms of recognized methodological procedures. Therefore, 257 sample size was considered adequate for providing unwavering parameter approximation and reliable testing. These 257 samples were selected using the stratified simple random approach, which ensures a proportional representation of gender and school distribution while staying random as shown in Figure 2. Stratified simple random sampling is the best sampling method for this study, as it preserves randomness while ensuring a proportionate representation of gender and school distribution. To ensure adequate representation of each category, the population is first stratified by gender and school. Simple random sampling is then used to choose participants within each stratum, ensuring equity and removing selection bias. With 196 males and 61 females, the final sample of 257 students produced by this method appropriately represents the target population’s gender distribution.

### 3.3. Measures and ethical considerations

In terms of ethical issues, Addis Ababa University’s ethics committee provided institutional ethical approval, under protocol number [76/2024]. All participants provided informed consent prior to data collection, in accordance with the principles of the Declaration of Addis Ababa University’s research guidelines. Then authorization was obtained from Addis Ababa Institute of Technology’s college dean to carry out study and gather data. After briefly describing the goal of the study and notifying the participants that their responses would be kept private, the researchers and 2 assistants delivered the premade questionnaire to the selected students in their classroom.

Data were acquired using a standardized self-administered questionnaire. It is divided into 4 parts: demographic information, social support, depression, and substance abuse-related items.

*Sociodemographic variables* includes, gender, religion, students’ intimate friend, romantic relationship status, or satisfaction in friendships and parent’s marital status, family occupation, monthly income level, and frequency of family drug use.

A pilot survey was conducted to assess the reliability and understandability of 3 main variables of the study. Some psychological measurement instruments, such as scales of depression, perceived social support, and substance use, have been adapted and tested within Ethiopian populations. The available empirical evidence indicates that these scales can be used appropriately in Ethiopian contexts, provided they are properly translated and reported. The pilot testing was conducted on 26 students who had comparable backgrounds to the main respondents but were not involved in the main study. Cronbach alpha was used to measure the scales’ reliability based on data obtained during the pilot survey. The Cronbach alpha result and general description of the 3 study variables in the pilot survey were as follows.

#### 3.3.1. Depression Scale

The Beck Depression Inventory (BDI) is the most widely used scale for diagnosing depression, and it was amended as BDI-II to be consistent with the Diagnostic and Statistical Manual of Mental Disorders.^[46]^ The scale consists of 21 items, each with 4 response choices of varying severity “over the past two weeks.” The total score reflects the degree of depression. The BDI-II has been widely investigated and is highly reliable and valid for assessing depression severity.^[13,46]^ Ethiopian and international researchers have examined the reliability and cultural appropriateness of commonly used depression or mental health screening instruments in education, clinical, and community settings.^[47–49]^ The present research found that the reliability coefficient for the Beck-II Depression Inventory was 0.89. This shows that the BDI-II is a highly dependable tool for diagnosing depressive symptoms in the current study area.

#### 3.3.2. Perceived social support

The 12-item Multidimensional Scale of Perceived Social Support^[50]^ was used to measure social support. This questionnaire evaluates the perceived sufficiency of social assistance from family (e.g., “I get the emotional help and support I need from my family”), friends (e.g., “I can count on my friends when things go wrong”), and special other person (e.g., “There is a special person who is around when I am in need”). Items are graded on a 5-point Likert scale from 1 (strongly disagree) to 5 (strongly agree). The mean total and subscale ratings range from 1 to 5, with higher values indicating more perceived social support. Previous studies have confirmed strong internal consistency and construct validity.^[47]^ This scale has been widely employed in Ethiopian students, typically reporting satisfactory internal consistency.^[50]^ In the present study, Multidimensional Scale of Perceived Social Support has good internal consistency, with a Cronbach α of.91 for the total scale.

#### 3.3.3. Substance Abuse Scale

The Alcohol, Smoking, and Substance Involvement Screening Test (ASSIST) was used to assess student use of substances.^[51]^ In 1997, an international group of drug misuse researchers developed the ASSIST in response to the need for an accurate and practical screening test for problematic or hazardous substance abuse that is also culturally appropriate. This scale has also been utilized in Ethiopian health study with satisfactory validity and reliability coefficient.^[50]^ There are 8 items. In this study, the Cronbach alpha coefficient for ASSIST was 0.91.

### 3.4. Data analysis

After the data was acquired by questionnaire, its completeness was verified, the data was entered and analyzed using SPSS version 25. The demographic features of the individuals were calculated using simple descriptive statistics (percentage and frequency). To investigate the relationship between social support, depression, and substance abuse, Pearson correlation was used. Moreover, a higher order multiple analysis of variance (MANOVA) was performed using socio-demographic characteristics such as parents’ marital status, family occupation, monthly income level, family drug use frequency, intimate friend, love relationship, and satisfaction in relationship with friend/s as predictor variables and depression and substance abuse as outcomes. Finally, structural equation modeling using LISREL 8.7 was used to study depression’s mediating function in the link between social support and substance abuse.

The statistical assumptions for structural equation modeling (normality, multicollinearity, linearity, and homoscedasticity) as well as preliminary analysis (missing values, errors, outliers, and skewed distribution) were examined prior to the main data analysis.^[52]^ The frequencies of the items were calculated in order to identify missing values. Less than 5% of the entire dataset’s missing values are acceptable. But there weren’t any missing values. The majority of the assumptions that are suggested by numerous sources are homoscedasticity, linearity, multicollinearity, and data normality.^[52,53]^ The data screening and preliminary analysis section addressed whether the data were normal. Furthermore, the regression residuals were examined and found to be within the suggested range of −2.5 to 1.75, indicating that the data met the assumption of normality.^[52]^ The correlation among variables, the variance inflation factor, and tolerance values were examined to verify multicollinearity among constructs and to prevent significant overlap among latent variables. High correlations between variables (*r* > .9) raise concerns about multicollinearity.^[54]^ In the present study, correlation coefficients among variables were below 0.72, indicating no overlap among the variables.^[50]^

## 4. Results

The data obtained from 243 Addis Ababa Institute of Technology final year regular undergraduate students was analyzed to arrive at specific conclusions on research questions. However, 14 students did not complete the questionnaire.

Among the total respondents in Table 1, 187 (77%) of the samples were males, the remaining 56 (23%) were females. The mean age of the participants was 23.74 with a standard deviation of 1.28. Out of the total participant 185 (76.1%) were orthodox religion followers followed by 32 (13.2%) Muslim, 13 (5.3%) protestant. Around 56.8% (n = 138) of participants were from rural residences, and the remaining 43.2% (n = 105) came from urban. 77%(n = 187) of participants responded that their parents were living together. The participants’ prominent family occupation was trade 39.9% (n = 97), and the second was farming 29.6% (n = 72). Among the overall respondents, 67.5% (n = 164) had intimate friend/s, the remains 32.5% (n = 79) did not have. The majority, 77.4% (n = 188) did not have boy/girlfriend, only 22.6% (n = 55) had boy/girlfriend. 77.8% (n = 189) were satisfied with their relationship with friends. 16.9% (n = 41) were not satisfied and 5.3% (n = 13) were very satisfied. Concerning the parents’ drug use frequency, 21.8% (n = 53) responded that their parents used alcohol and other drugs more than 3 times a week, 14% (n = 34) twice a week, 18.1% (n = 44) once a week, and 18.1% (n = 44) daily. The remaining 28% (n = 68) responded that their parents did not use substance/s in their whole.

**Table 1:** Sociodemographic features of students.

Variables	N	%	Variables	N	%
Gender			Intimate friend		
Male	187	77	Yes	164	67.5
Female	56	23	No	79	32.5
Religion			Monthly income		
Orthodox	185	76.1	Very low	50	20.6
Muslim	32	13.2	Low	84	34.6
Protestant	13	5.3	Medium	76	31.5
Catholic	11	4.5	High	29	11.9
Others*	2	.80	Very high	4	1.6
Ethnic origin			Family occupation		
Tigray	47	19.3	Merchant	97	39.9
Amhara	75	30.9	Gov’t employee	32	13.2
Oromo	63	25.9	NGO employee	18	7.4
SNNP	35	14.4	Daily laborer	2	0.8
Others**	23	9.5	Private employee	18	7.4
			Farmer	72	29.6
			Other***	4	1.6
Residence			Love relationship		
Rural	138	56.8	Yes	55	22.6
Urban	105	43.2	No	188	77.4
Parents’ marital status			Satisfaction in r/ship with friends		
Live together	187	77.0	Very satisfied	13	5.3
Separated	8	3.3	Satisfied	189	77.8
Divorced	13	5.3	Not satisfied	41	16.9
Mother passed away	8	3.3			
Father passed away	16	6.6			
Both parental loss	11	4.5			
			Parents drug use frequency		
			Never in my whole	68	28
			Once a week	44	18.1
			Twice a week	34	14
			> 3 times a week	53	21.8
			Daily	44	18.1

N.B: N-Number.

* Non-religion.

** House wife.

*** Benishangul, Afar and Somalia.

### 4.1. The effect of socio-demographic factors on the combined dependent variables (depression and substance abuse)

The effect of relationship variables (intimate friend, romantic relationship status, or satisfaction in friendships) and parents’ marital status, family occupation, monthly income level, and family drug use frequency on the combined dependent variables (depression and substance abuse) were presented in Tables 2 to 5.

**Table 2:** MANOVA test between predictor variables (parent’s marital status, family occupation, monthly income level, and family drug use frequency) and dependent measures (depression and substance abuse).

Predictors name	DVs	Test	Value	F	H. df	E. df	Sig.	PES
Parents’ marital Status (A)	Substance abuse Depression	Wilks’	.81	2.69	10.00	242.0	.004	.10
Family occupation (B)	Substance abuse Depression	Wilks’	.95	.58	12.00	242.0	.857	.03
Monthly income (C)	Substance abuse Depression	Wilks’	.80	3.50	8.00	242.0	.001	.10
Family drug use frequency (D)	Substance abuse Depression	Wilks’	.87	2.17	8.00	242.0	.031	.07
A × B Depression	Substance abuse	Wilks’	.82	2.11	12.00	242.0	.07	.10
A × C Depression	Substance abuse	Wilks’	.86	1.87	10.00	242.0	.050	.07
A × D Depression	Substance abuse	Wilks’	.83	2.36	10.00	242.0	.051	.09
B × C Depression	Substance abuse	Wilks’	.78	1.36	24.00	242.0	.129	.12
B × D Depression	Substance abuse	Wilks’	.68	1.49	34.00	242.0	.058	.17
C × D Depression	Substance abuse	Wilks’	.81	1.07	26.00	242.0	.383	.10
A × B × C Depression	Substance abuse	Wilks’	1.00	.	.00	121.5	.	.
A × B × D Depression	Substance abuse	Wilks’	1.00	.	.000	121.5	.	.
A × C × D Depression	Substance abuse	Wilks’	1.00	.	.000	121.5	.	.
B × C × D Depression	Substance abuse	Wilks’	.76	1.38	26.00	242.0	.109	.13
A × B × C × D Depression	Substance abuse	Wilks’	1.00	.	.000	121.5	.	.

E = df-error degrees of freedom, H = df-hypothesis degrees of freedom, MANOVA = multiple analysis of variance, PES = partial eta squared.

As observed from Table 2 above, a 4-way between-subjects MANOVA was conducted on 2 dependent variables: depression and substance abuse. From Table 2, it is observed that parents’ marital status had a significance difference on combined dependent variables, Wilk Lambda = .81, *F* (10,242) = 2.70, *P* < .05, PES = .10. There was also a statistically significant difference in monthly income level on combined dependent variables, Wilk’s Lambda, .80, *F* (8242) = 3.50, *P* < .001, PES = .10. In addition, family drug use frequency was statistically significant, Wilk’s Lambda, .87, *F* (8242) = 2.17, *P* < .05, PES.10. No statistically significance difference on family occupation, *F* (12,242) = .58, *P* > .05 were observed on the 2 combined dependent measures. A multivariate interaction of predictor variables, Parents’ marital status × monthly income level, was found statistically significant, Wilk’s Lambda, .86, *F* (10, 242) = 1.87, *P* < .05, PES = .07. However, the rest of the interaction effects were found nonsignificant in a multivariate test, all at *P > .05.*

According to Table 3, parents’ marital status and monthly income had a substantial impact on student substance abuse and depression. Those who lost both parents and had extremely low income had the highest rates of substance abuse (*M* = 36.67) and depression (*M* = 21.89). Students whose parents lived together and earned more money reported the lowest levels of both concerns. There were also significant interaction effects between marital status and location, as well as income and family occupation, on substance abuse and depression. Family drug usage was significant in multivariate analyses but not in individual outcome indicators. Overall, social and economic variables significantly influenced students’ mental health and substance abuse behaviors.

**Table 3:** Univariate test for parent’ marital status and monthly income level on each dependent measure.

Predictors	Dependent variable	SS	Df	MS	F	*P*	PES
Parents’ marital Status (A)	Substance abuse	1389.83	5	277.97	2.33	.046	.09
	Depression	742.72	5	148.54	4.16	.002	.15
Family Occupation (B)	Substance abuse	422.43	6	70.41	.59	.738	.03
	Depression	162.44	6	27.07	.76	.604	.04
Monthly income (C)	Substance abuse	2934.83	4	733.71	6.15	.000	.17
	Depression	619.98	4	154.99	4.35	.003	.13
Family drug Use frequency (D)	Substance abuse	957.37	4	239.34	2.01	.098	.06
	Depression	256.48	4	64.12	1.80	.134	.06
A × B	Substance abuse	1369.34	6	228.22	1.91	.084	.09
	Depression	149.77	6	24.96	.70	.650	.03
A × C	Substance abuse	1936.83	5	387.37	3.25	.009	.12
	Depression	382.55	5	76.51	2.15	.065	.08
A × D	Substance abuse	1442.59	5	288.52	2.42	.040	.09
	Depression	450.74	5	90.15	2.53	.033	.09
B × C	Substance abuse	2658.26	12	221.52	1.86	.046	.15
	Depression	825.47	12	68.79	1.93	.037	.16
B × D	Substance abuse	2781.18	17	163.60	1.37	.162	.16
	Depression	1234.85	17	72.64	2.04	.014	.22
C × D	Substance abuse	1966.05	13	151.24	1.27	.242	.12
	Depression	768.12	13	59.09	1.66	.079	.15
A × B × C	Substance abuse	.000	0	.	.	.	.00
	Depression	.000	0	.	.	.	.00
A × B × D	Substance abuse	.000	0	.	.	.	.00
	Depression	.000	0	.	.	.	.00
A × C × D	Substance abuse	.000	0	.	.	.	.00
	Depression	.000	0	.	.	.	.00
B × C × D	Substance abuse	1908.85	13	146.835	1.231	.266	.12
	Depression	673.998	13	51.846	1.454	.145	.13
A × B × C × D	Substance abuse	.000	0	.	.	.	.00
	Depression	.000	0	.	.	.	.00

DF = degrees of freedom, MS = mean sum of squares, PES = partial eta squared, SS = sum of squares.

The above a multivariate test Table 4 is a 3-way between-subjects MANOVA table which was performed on the 2 combined dependent variables: depression and substance abuse. A multivariate test shows a statistically significant effect of satisfaction in relationship with friend/s on the 2 combined dependent variables; however, love relationship as a predictor variable for depression and substance abuse was not statistically significant, *P > *.05. None of the interactions of the predictor variables were significant.

**Table 4:** MANOVA test between predictor variable (intimate friend, relationship status [lover], and satisfaction in relationship with friends) and dependent measures (depression and substance abuse).

Predictors	Dependent variable		Value	F	H. df	E. df	*P*	PES
Intimate friend (A)	Substance abuse Depression	Wilks’	.97	3.13	2.00	230.0	.045	.03
Lover (B)	Substance abuse Depression	Wilks’	.98	2.78	2.00	230.0	.064	.02
Satisfaction in r/ship with friends (C)	Substance Abuse Depression	Wilks’	.96	2.39	4.00	460.0	.050	.02
A × B	Substance Abuse Depression	Wilks’	.99	.67	2.00	230.0	.514	.01
A × C	Substance abuse Depression	Wilks’	.96	2.4	4.00	460.0	.053	.02
B × C	Substance abuse Depression	Wilks’	.99	.64	4.00	460.0	.637	.01
A × B × C	Substance abuse Depression	Wilks’	.97	1.7	4.00	460.0	.160	.01

E = df-error degrees of freedom, H = df-hypothesis degrees of freedom, MANOVA = multiple analysis of variance, PES = partial eta squared.

As displayed in Table 5 above, having or not having intimate friend/s had a significant effect on the variation of substance abuse. Depression had a variation in an intimate friend. Moreover, satisfaction in relationships with friend/s had a significant effect on substance abuse and on depression. Love relationship was found nonsignificant with substance abuse and with depression. Post hoc multiple comparison test identified which group (either those who had an intimate friend/s or not) abused substances and were highly depressed. The study found that participants who did not have intimate friend/s had statistically significantly higher substance abuse (*M* = 23.21, SE = 3.41) than those who did have intimate friends (*M* = 13.16, SE = 2.49), and those who had no intimate friends were found highly depressed than their counterparts (*M* = 16.09, SE = 1.73). The post hoc comparison test revealed that participants who were not satisfied with their relationship with friend/s (*M* = 26.48, SE = 3.59) were significantly higher substance abusers and depressed (*M* = 25.13, SE = 3.62).

**Table 5:** Univariate ANOVA test for intimate friend and satisfaction in relationship with Friend/s (predictor variables) on each dependent measure.

Predictors	Dependent variable	SS	df	MS	F	*P*	PES
Intimate friend (A)	Substance abuse	792.61	1	792.61	5.67	.024	.02
	Depression	239.27	1	239.27	4.93	.027	.02
Lover (B)	Substance abuse	70.59	1	70.59	.46	.500	.00
	Depression	1.67	1	1.67	.03	.853	.00
Satisfaction in r/ship with friends (C)	Substance abuse	1453.97	2	726.99	4.71	.010	.04
	Depression	907.93	2	453.96	9.36	.000	.08
A × B	Substance abuse	57.73	1	57.73	.37	.542	.00
	Depression	63.44	1	63.44	1.31	.254	.01
A × C	Substance abuse	1006.37	2	503.18	3.26	.040	.03
	Depression	400.01	2	200.00	4.12	.017	.03
B × C	Substance abuse	126.02	2	63.01	.41	.665	.00
	Depression	101.36	2	50.68	1.04	.354	.01
A × B × C	Substance abuse	810.88	2	405.44	2.63	.075	.02
	Depression	279.60	2	139.80	2.88	.058	.02

DF = degrees of freedom, MS = mean sum of squares, PES = partial eta squared, SS = sum of squares.

### 4.2. The correlation between social support, depression, and substance abuse

The Pearson correlation in Table 6 revealed substantial correlations between depression, substance abuse, and social support. Depression and substance abuse had a significant positive correlation (*r* = .72, *P* < .01). Higher levels of depression are linked to a higher risk of substance use among students. Depression and social support had a negative connection (*r* = −.35, *P* < .01), suggesting that lower levels of depression are associated with stronger perceived social support. The moderate negative relationship between social support and substance abuse (*r* = −.46, *P* < .01) suggests that those who feel supported are less likely to use substances as a coping mechanism. This supports the idea that strong, meaningful connections might help people avoid damaging ways of dealing with psychological sadness and instead foster persistence.

**Table 6:** The relationships between depression, substance abuse, and social support.

Variables	Depression	Substance abuse	Social support
Depression	1		
Substance abuse	.72**	1	
Social support	−.35**	−.46**	1

** Correlation is significant at the 0.01 level (2-tailed).

### 4.3. The mediating role of depression in the association between social support and substance abuse

**Figure 3.**
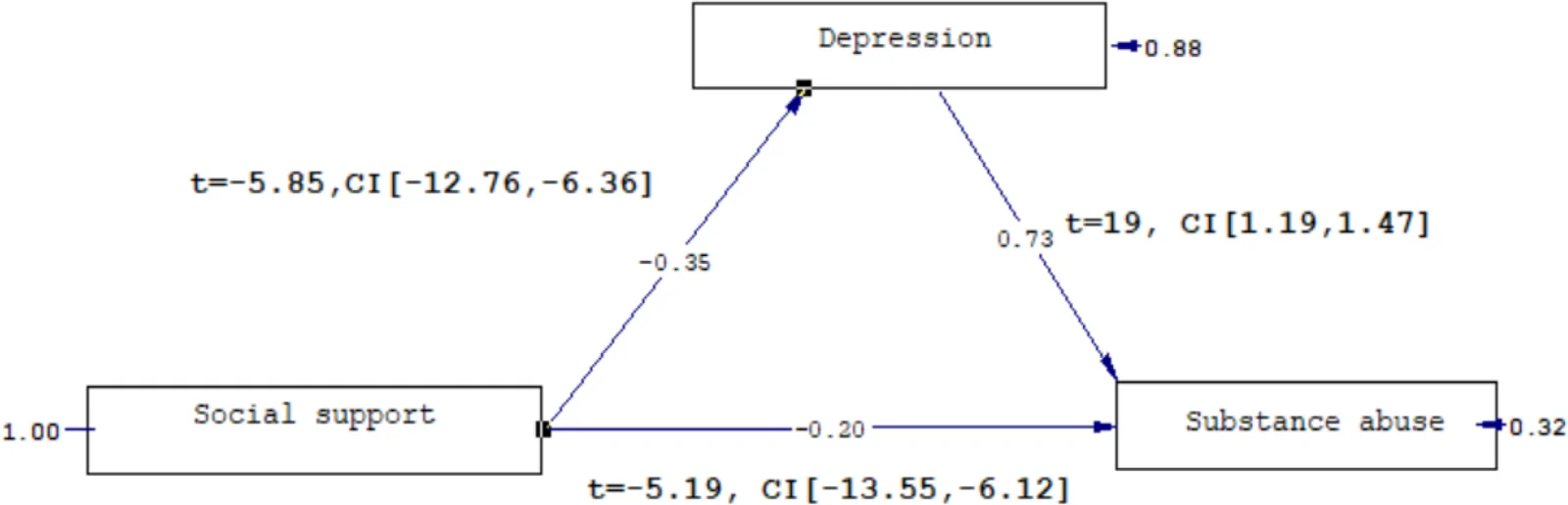
Verified conceptual framework of the study. Path coefficients, *t*-values, and confidence intervals demonstrate the strength of the correlations between depression, drug addiction, and social support.

Social support does more than merely buffer depression; it also helps students avoid depression, a known risk factor for substance abuse. The mediating role of depression in the association between social support and substance abuse was presented in Figure 3.

Figure 3 and Table 7 revealed that higher social support tends to experience lower levels of depression. The beta value here is –0.35, indicating that a meaningful drop in depression is associated with better social support. This relationship is statistically strong and reliable, as shown by a *t*-value of –5.85 and a confidence interval that clearly stays below zero (–12.76 to–6.36), reinforcing the negative connection. Depression is strongly linked to substance abuse (β = 0.73, *t* = 19, CI [1.119 to 1.47]). The positive standardized beta showed indicated a large positive effect of depression on substance use. However, even far from its link to depression, social support also directly reduces substance abuse. This is reflected in the beta value of –0.20 at *t* = –5.19, CI [–13.55 to –6.12], which is also significant. Regarding model fit indices, the data and the model fit each other well in the current Structural equation model (χ2/df = 2.12), which yielded a significant result at *P* < .01. Furthermore, as evidenced by CFI = 0.92 and TLI = .91, RMSEA = .039, and RMR = .042, the data fit the model well.

**Table 7:** Standardized path coefficients and explained variance.

Pathway	β (Standardized)	Indirect effect	Total effect	*t*-Value	95% CI
Social Support → Depression	–0.35	–	–0.35	–5.85	[–12.76 to –6.36]
Depression → Substance Abuse	0.73	–	0.73	19.00	[1.19 to 1.47]
Social Support → Substance Abuse	–0.20	–	–0.20	–5.19	[–13.55 to –6.12]
Social Support → Depression → Substance Abuse	–0.20	–0.25	–0.45	–	–

As shown in Table 7 and Figure 3, to measure how much of the effect works *through* depression (the indirect effect), we multiply the beta values for the 2 steps: Indirect effect = –0.35 × 0.73 = –0.25. When we combine this with the direct effect (–0.20), we get the total effect of social support on substance abuse: Total effect –0.20 + (–0.25) = –0.45. This total shows that overall, social support has a moderate-to-strong effect on reducing substance abuse, and a good portion of that happens through lowering depression. The model shows that 45% of the differences in substance abuse levels can be explained by how much social support a person has. In short, students’ perceived social support play a significant role in lowering depression, and that in turn helps reduce substance abuse. Depression has a mediating role in the relationship between social support and substance abuse. However, social support also has a direct benefit – helping people stay away from substance use, even apart from its effect on mood. These findings suggest that investing in supportive relationships and communities could be a powerful way to promote both mental health and reduce risky behavior like substance abuse.

## 5. Discussion

The current study found substantial links between perceived social support and high-risk substance abuse behaviors and depressed symptoms among higher education students. Although previous research has looked at the link between social support and abuse of drugs, our study expands on the findings to include the impact of depression in the correlation between social support and substance misuse behaviors in the higher education population. Lazarus and Folkman^[2]^ stress and coping theory helps understanding the influence of perceived social support on depression and substance abuse in the context of this study.

The current study’s first findings indicate that parents’ marital status and monthly income level are important predictors for substance dependence as well as depression among adolescents. This finding show that adolescents from non-intact households (parents who are divorced or separated) are more likely to have psychological discomfort and behavioral problems. In line with the present study, existing research consistently shows that parental separation can impair emotional development and increase the possibility of internalizing and externalizing behaviors in children.^[55,56]^ The present findings also highlight the negative impact of economic stress on adolescents’ mental wellbeing. Lower-income families frequently face ongoing strain, restricted access to health and educational possibilities, and higher exposure to neighborhood danger factors – all of which could contribute to the prospect of substance abuse and symptoms associated with depression.^[57,58]^ The strong interaction effects also revealed that adolescents from low-income, non-intact homes were exposed to substance abuse. This is consistent with ecological theories of development, which highlight the role of multiple interconnected risk factors in shaping what happens to students.^[59,60]^

There is a negative relationship between depression and social support, meaning that lower levels of depression are associated with greater perceived social support. Social support is a crucial factor in reducing depression among students. In line with the present study, according to the several studies, students with low-quality social support are more likely to experience depression symptoms.^[3,14–16,19,20]^ Local studies at Dilla and Debre Markos Universities in Ethiopia likewise indicated that students who thought they had little or no assistance were substantially more likely to have moderate to severe depression.^[23,24]^ In similar vein, people who start drinking too much alcohol at a young age and later vulnerable to depression.^[4]^ According to an integrative perspective, social support can indirectly lower the likelihood of substance abuse by lowering psychological distress and depression. Another finding also highlighted the significance of establishing robust support networks, such as peer and parental support in educational settings, in order to stop depression.^[5–8]^ The present findings are consistent with Lazarus and Folkman^[2]^ stress and coping theory, which highlights how social support may help reduce the emotional impact of stressful events. This finding demonstrated that when university students feel supported by their family, friends, or classmates, they are better able to cope with the stresses of campus life. Strong familial and social bonds are commonly seen as a manifestation of resilience in Ethiopia. However, for many students, specifically those studying away from home, increasing social support within higher education institutions may be an effective way to help them feel less melancholy and more resilient to emotions.

The present investigation found that greater levels of depression are associated with an increased probability of substance abuse among students. These finding was further corroborated in many investigations.^[33–37]^ Similarly, there is evidence that persons who are depressed may be more prone to developing a substance misuse issue early in life.^[39]^ Psychological conditions such as stress, anxiety, and depression at baseline posed significant potential hazards for the development of alcohol, nicotine, and illicit drug dependence and abuse throughout the follow-up period.^[21,40,41]^ Congruently, among Canadian postsecondary students, cannabis and tobacco use were significantly associated with depressed symptoms, which is consistent with the current study and suggests a tendency to self-medicate psychological suffering.^[33]^ A study among Nigerian medical students^[34]^ further supported this connection in diverse student populations. Joel research indicates that mental health conditions at baseline posed significant risks for the development of illicit substance abuse and dependence, as well as alcohol and nicotine, during the follow-up period.^[40]^ Significant and long-lasting correlations were discovered between specific mood and anxiety disorders, behavioral disorders, substance use history, and other conditions.^[21,41]^ In line with stress and coping theory, these results revealed that proactive coping, such as good social support, predicted improved well-being. Interestingly, according to stress and coping theory,^[2]^ social support roles are a coping resource that mitigates the psychological effects of stressors. By decreasing depression signs and improving adaptive coping strategies, social support diminishes the possibility of substance use actions that appear as a maladaptive mechanism for handling emotional distress. Social support has an immediate effect and indirectly affects depression on substance abuse. According to the path diagram model of the study, 45% of the variance in substance abuse levels may be explained by a person’s perceived social support. According to previous research, having social support commonly prevents the adoption of hazardous coping mechanisms, such as substance addiction.^[14,25,26]^ Similarly, peer environment predicts tobacco, alcohol, and drug use.^[27–31]^ Furthermore, there is a significant beneficial relationship between adolescent substance abuse and social support. Students select classmates based on social group norms, and over time, the peer group may be conditioned to comply. Adolescents may, for example, associate with their substance-using peers because they believe that substance abuse is the norm and may alter their behavior to match this social setting.^[32]^ A favorable correlation may be expected in situations when peer-connecting social venues (such as clubs, sports teams, and friendship groups) also provide easy access to drugs.^[27,28]^ Depression has a mediating function in the link between social support and substance abuse. In addition to its influence on mood, social support has a direct benefit: it helps people avoid substance abuse. These findings show that investing in supportive relationships and communities might be an effective method to increase mental health while also reducing dangerous behavior such as substance abuse.

## 6. Conclusions, implications, and limitations

The core of the present study is a simple yet profound truth: having someone to rely on may make all the difference. According to the findings, when adolescents feel supported by family, friends, or mentors, they are less likely to experience depression. When depression is controlled, the desire to escape through drugs or alcohol decreases. Furthermore, the current study found that parents’ marital status and monthly income level are key predictors of substance abuse disorder and depression in higher education students.

This result has both theoretical and practical implications. First and foremost, this study contributes theoretical evidence to the current literature on the function of depression in the link between substance abuse and social support. Second, the findings have significant management implications for understanding and overseeing characteristics that may contribute to greater levels of depression and substance abuse among students. The current study found that social support has a vital impact in reducing depression, which helps to minimize substance abuse. This is true because strong familial and social bonds are widely used in Ethiopian culture to show resilience. However, for many students, particularly those studying away from home, increasing social support at university may be an effective technique for feeling less depressed and more resilient to emotions. This study recommends that, for better results, the university should improve its support system and empower specialists who work on students’ mental health, such as therapists and psychologists, by establishing its own working center. As a consequence, these professionals in the region will provide regular counseling and facilitate group treatment, especially for persons recognized as at risk so that they may learn from one another through experience sharing. In summary, making a young person feel supported does more than improve their mood; it may transform their life. It is not only about avoiding drugs and alcohol; it’s about laying an emotional foundation that will allow them to succeed no matter what obstacles they face.

When interpreting these findings, it is critical to consider a variety of the present study limitations. First and foremost, because our sample mainly consisted of Addis Ababa University students, our findings may not be applicable to other populations. Second, we employed self-report as our measure of substance abuse, which may have reduced the accuracy of the data collected on substance use behaviors. Other research has found that, rather than obtaining information directly from peers, young people’s views of their friends’ antisocial behavior are a stronger predictor of their engagement in hazardous activity. Future study should look at broader friend networks to see whether these outcomes vary based on the size of an adolescent’s friend network. Third, the cross-sectional technique utilized in this study limits extrapolations about causal relationships. Future researchers who are interested are advised to undertake a longitudinal study to evaluate the association between depression and substance abuse in the university student population. Fourth, 45% of the variance was explained by depression level and perceived social support, indicating that 55% of the variance was explained by unmeasured or residual confounding factors (e.g., contextual, behavioral, or socio-demographic variables not included in the model) that may have affected the observed associations. Future longitudinal or experimental studies are recommended to add more variables and reduce potential confounding, and enrich causal inference.

## Acknowledgments

I am grateful to the participants for their participation in this study.

## Author contributions

**Conceptualization:** Kelemu Zelalem Berhanu, Kefie Manaye Mengistie.

**Data curation:** Kelemu Zelalem Berhanu, Kefie Manaye Mengistie.

**Formal analysis:** Kefie Manaye Mengistie.

**Project administration:** Kefie Manaye Mengistie.

**Supervision:** Kefie Manaye Mengistie.

**Validation:** Kelemu Zelalem Berhanu.

**Writing – original draft:** Kelemu Zelalem Berhanu.

**Writing – review & editing:** Kelemu Zelalem Berhanu.
